# A Unique Case of Pregnancy-Associated Spontaneous Coronary Artery Dissection Presenting As Generalized Tonic-Clonic Seizure

**DOI:** 10.7759/cureus.69642

**Published:** 2024-09-18

**Authors:** Saad Ur Rahman, Seemab Imtiaz Gill, Muhammad U Rana

**Affiliations:** 1 Internal Medicine, Carle Illinois College of Medicine, Urbana, USA; 2 Internal Medicine, Carle Foundation Hospital, Urbana, USA; 3 Internal Medicine, King Edward Medical University, Lahore, PAK

**Keywords:** generalized tonic clonic seizures, postpartum, scad in pregnancy, st-elevation myocardial infarction (stemi), ventricular fibrillation (vf) storm

## Abstract

Spontaneous coronary artery dissection (SCAD) is an uncommon form of non-atherosclerotic coronary artery disease, characterized by a sudden tear in coronary artery layers. Pregnancy-associated SCAD (P-SCAD) is a rare variant occurring during pregnancy or postpartum. We present a case of P-SCAD in a 31-year-old postpartum female who experienced generalized tonic-clonic seizures along with ventricular fibrillation. Despite its rarity, P-SCAD should be considered in postpartum females with atypical symptoms, such as seizures or ventricular fibrillation. This case underscores the need for heightened clinical suspicion and timely intervention to manage P-SCAD effectively. Early recognition can lead to improved patient outcomes and appropriate management strategies, ranging from conservative approaches to percutaneous interventions or implantable cardioverter-defibrillator placement.

## Introduction

Spontaneous coronary artery dissection (SCAD) is a rare yet life-threatening condition involving arterial wall separation, causing reduced myocardial blood flow. Pregnancy-associated SCAD (P-SCAD) is even rarer, posing challenges due to maternal-fetal considerations [1,2]. Unusual presentations like generalized tonic-clonic seizures have emerged, complicating diagnosis and risking maternal-fetal health [3,4].

We report a case of P-SCAD in a patient who presented with generalized tonic-clonic seizures. By analyzing this specific case, we aim to highlight the importance of early recognition, comprehensive evaluation, and multidisciplinary management in such instances. Additionally, we will discuss the current literature on P-SCAD and its association with neurological symptoms.

## Case presentation

A 31-year-old G2P2A0 woman with no notable medical history presented to the emergency department following a generalized tonic-clonic seizure at home. She reported ongoing post-ictal chest pain for the past hour and was 10 days postpartum from a cesarean section. Shortly after her initial assessment, she experienced another episode of generalized tonic-clonic seizure. Her rhythm on telemetry turned to ventricular fibrillation with loss of pulse on subsequent examination. Two rounds of cardiopulmonary resuscitation and defibrillation were performed, successfully restoring spontaneous circulation (ROSC).

**Figure 1:**
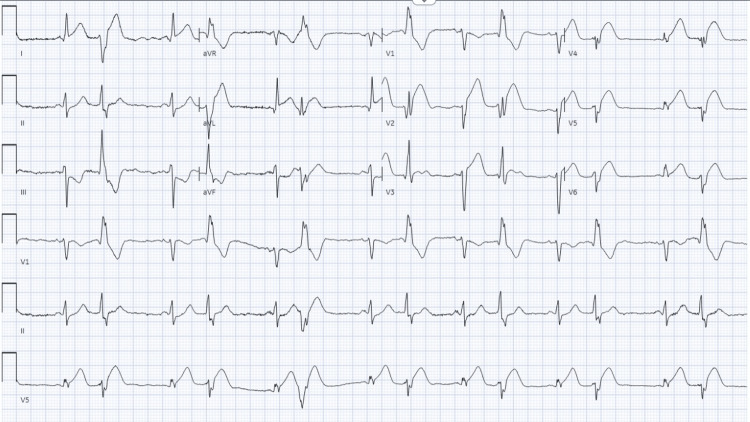
Initial EKG on presentation remarkable for ST-segment elevation in leads I, AVL, V2, and V3 on EKG EKG: electrocardiogram

Post-ROSC, the electrocardiogram (EKG) showed ST-segment elevation myocardial infarction (STEMI) in anterolateral leads with the most significant changes in leads V2, V3, I, and AVL (Figure 1). High-sensitivity troponin levels were elevated to >30000.

Urgent CT brain and CTA chest scans were unremarkable. The patient was administered aspirin and placed on a heparin drip, reporting improvement in chest pain. Nevertheless, due to the atypical presentation of seizures and STEMI in the postpartum period, the patient underwent emergent left heart catheterization for further evaluation.

**Figure 2:**
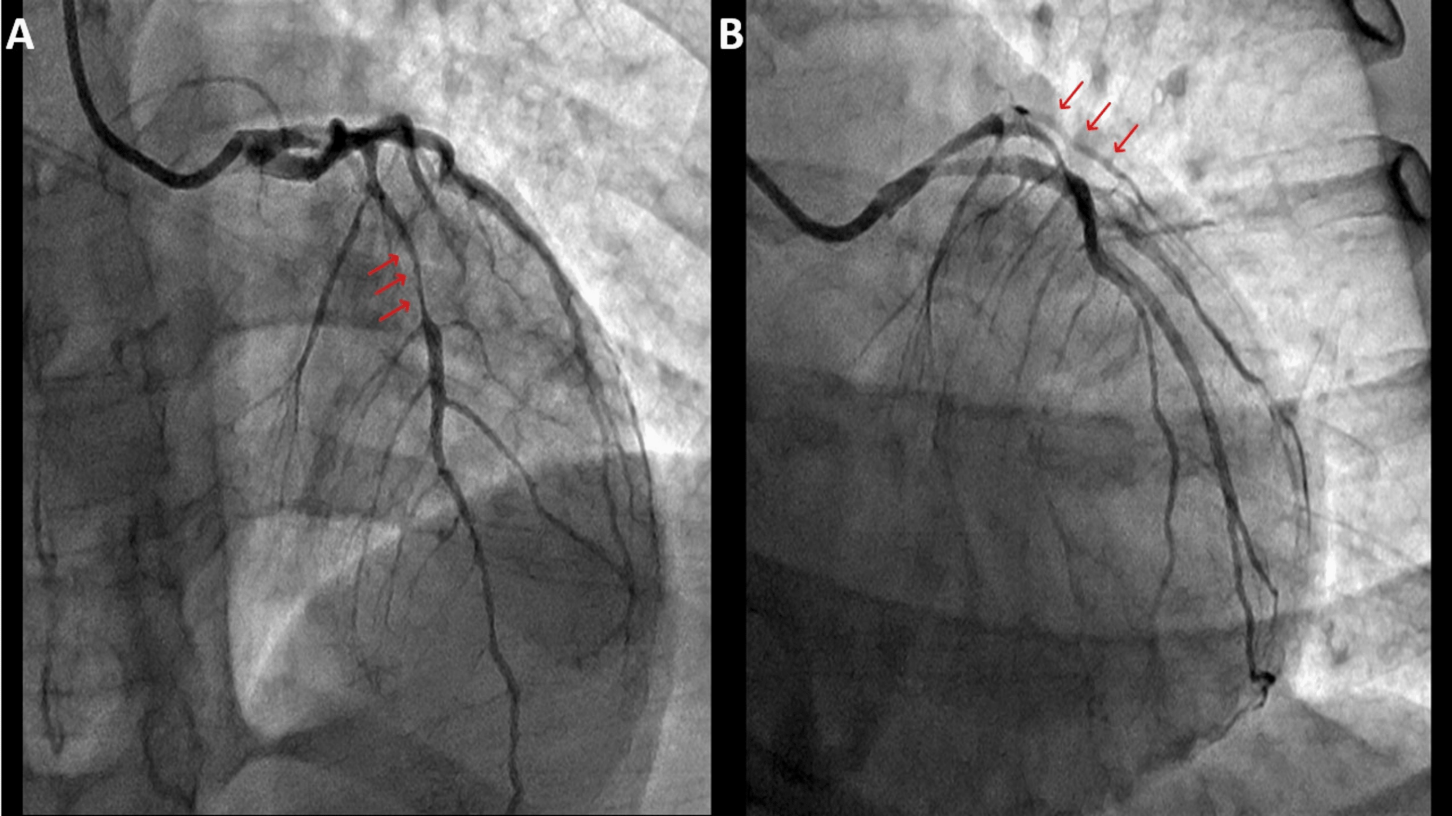
Red arrows pointing toward the coronary artery dissection in proximal LAD extending into the mid LAD (A - AP cranial view/coronary angiogram) and involvement of the first diagonal artery/D1 (B - LAO cranial view/coronary angiogram)​ LAD: left anterior descending artery, AP: anteroposterior, LAO: left anterior oblique

A coronary angiogram revealed SCAD involving the proximal left anterior descending artery (LAD) extending into the proximal to mid-LAD and the first diagonal artery (Figure 2).

**Figure 3:**
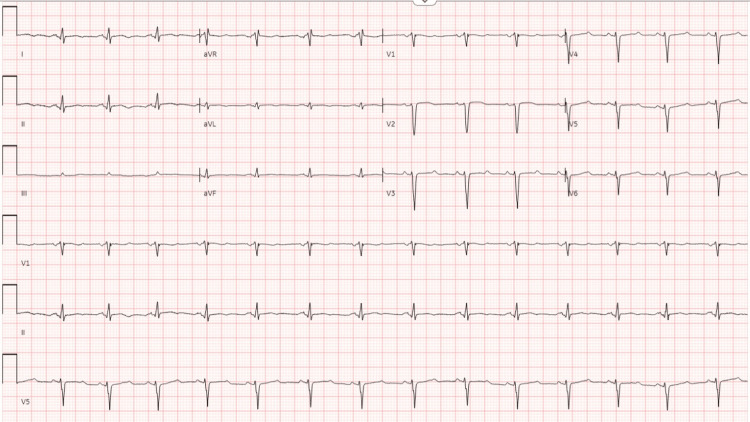
Repeat EKG in 24 hours showed significant improvement with medical management EKG: electrocardiogram

The case was discussed with the interventional cardiology team. Due to the complex site of coronary artery dissection, a conservative management approach was chosen, as additional invasive procedures risked exacerbating the dissection. The patient remained hemodynamically stable with minimal symptoms. Repeat EKG in 24 hours also revealed the resolution of the prior findings of ST elevation (Figure 3). She was discharged in stable condition on dual antiplatelet therapy, a beta-blocker, and a life vest. As the patient survived sudden cardiac arrest, a follow-up appointment was scheduled in four weeks for the placement of an automatic implantable cardioverter-defibrillator (AICD) as secondary prevention to guard against future episodes of cardiac arrhythmias. At the four-week follow-up, the patient remained asymptomatic and successfully underwent AICD placement. Medical management was continued with close monitoring.

## Discussion

P-SCAD presenting as generalized tonic-clonic seizures is a scarce and critical clinical scenario that poses diagnostic challenges and demands urgent multidisciplinary management. Current literature reports P-SCAD incidence as approximately 1.81 per 100,000 pregnancies [5]. This unique manifestation of SCAD during pregnancy can lead to delays in appropriate intervention, potentially jeopardizing the health and well-being of both the mother and the fetus.

The pathophysiological mechanisms leading to SCAD during pregnancy are not fully understood. Hormonal changes, particularly fluctuations in estrogen and progesterone levels, may weaken arterial walls, increasing susceptibility to dissection [1]. Increased cardiac output and vascular remodeling during pregnancy might also contribute [6]. The association of SCAD with generalized tonic-clonic seizures presents significant diagnostic challenges. Overlapping neurological symptoms may lead to misdiagnosis, delaying treatment. Physicians should consider SCAD as a potential cause in pregnant women presenting with seizures, especially those without a history of epilepsy or neurological disorders.

To ensure optimal outcomes for both the mother and the fetus, early recognition of P-SCAD is crucial. Access to advanced cardiac imaging modalities, such as coronary angiography or intravascular ultrasound, can aid in confirming the diagnosis and assessing the extent of coronary artery involvement [2].

The treatment approach for P-SCAD requires careful evaluation, with options including medical therapy, percutaneous coronary intervention (PCI), or coronary artery bypass grafting (CABG) depending on the severity and location of the dissection. Recent data shows that over 70% of conservatively managed patients experienced healing of SCAD lesions [7]. Conservative management involving aspirin and beta-blockers is recommended, often with a three- to five-day inpatient monitoring period. Revascularization is considered for ongoing/recurrent ischemia, hemodynamic instability, or left main dissection when conservative methods are insufficient. CABG is reserved for severe cases, especially with large-vessel dissections and hemodynamic compromise [8].

PCI for SCAD has shown lower success rates and more complications compared to atherosclerotic CAD treatment. SCAD-affected arteries are susceptible to iatrogenic dissections during PCI, potentially worsening vessel obstruction [9]. Decisions for surgery or PCI should prioritize both maternal and fetal well-being, guided by a risk-benefit assessment that considers the severity of ongoing symptoms and any hemodynamic compromise [10].

## Conclusions

P-SCAD, a rare factor in non-atherosclerotic myocardial infarction, can exhibit itself as a generalized tonic-clonic seizure. Vigilance toward the possibility of P-SCAD is of paramount importance, particularly in postpartum young women encountering symptoms like chest pain, seizures, or ventricular fibrillation. Prompt identification of pregnancy-related SCAD is essential to ensure the best possible results for both maternal and fetal well-being.
